# The behavioural phenotype of SATB2-associated syndrome: a within-group and cross-syndrome analysis

**DOI:** 10.1186/s11689-022-09426-0

**Published:** 2022-03-29

**Authors:** Stacey Bissell, Chris Oliver, Joanna Moss, Mary Heald, Jane Waite, Hayley Crawford, Vishakha Kothari, Lauren Rumbellow, Grace Walters, Caroline Richards

**Affiliations:** 1 School of Psychology, University of Birmingham, Edgbaston, Birmingham, UK; 2 School of Psychology, University of Surrey, Guildford, Surrey, UK; 3 Blackpool Teaching Hospitals NHS Foundation Trust, Blackpool, Lancashire, UK; 4 School of Health and Life Sciences, Aston University, Birmingham, UK; 5 Mental Health and Wellbeing Unit, Warwick Medical School, University of Warwick, Coventry, UK

**Keywords:** Behavioural phenotype, SATB2-associated syndrome, Autism, Angelman syndrome, Challenging behaviour, Repetitive behaviour, Stereotyped behaviour, Compulsive behaviour, Emotional characteristics

## Abstract

**Background:**

SATB2-associated syndrome (SAS) is a multisystem neurodevelopmental disorder characterised by intellectual disability, speech delay, and craniofacial anomalies. Although the clinical presentation of SAS is well-delineated, behaviours associated with SAS are less well-defined. Given the varied social profile reported in SAS of a ‘jovial’ predisposition and autistic behaviours, there may be phenotypic overlap with both Angelman syndrome (AS) and non-syndromal autism. This study aimed to describe behaviours in SAS in relation to chronological age and level of ability and contrast aspects of the behavioural phenotype with AS and non-syndromal autism.

**Methods:**

Informant report questionnaire measures of behaviour, emotion, and autism characteristics were completed for 81 individuals with SAS (aged 1–36 years; 43 male). Within-group associations were analysed, and categorical data were compared between pre-school (1–5 years), school-age (6–15 years), and adolescent and adult SAS sub-groups (16 years and over). Cross-syndrome subscale and item-level analyses were conducted for 63 individuals with SAS (aged 1–27 years; 31 male), who were matched according to age and level of ability to 63 individuals with AS (aged 2–25 years; 32 male) and 63 individuals with non-syndromal autism (aged 3–26 years; 53 male).

**Results:**

In SAS, higher rates of overactivity were moderately associated with lower self-help ability, and higher general anxiety scores were reported for males compared with females. Cross-syndrome subscale analyses uncovered several significant differences (*p* < .01), with comparatively low rates of stereotyped behaviour, overactivity, insistence on sameness and positive affect, and comparatively greater interest and pleasure and compulsive behaviour in individuals with SAS. Item-level analyses revealed a distinct profile of repetitive and autistic behaviours.

**Limitations:**

Developmental analysis was based on a cross-sectional rather than a longitudinal research design, the contribution of pain and sleep to behaviour was not explored, and molecular genetic testing to determine genotype–phenotype behavioural relationships was not possible.

**Conclusions:**

This study highlights the importance of behavioural comparisons to well-delineated groups and the utility of fine-grained item-level analyses to elucidate aspects of behaviour that might be syndrome related or shared across neurodevelopmental disorders. Future research is needed to further describe the distinctive repetitive and autistic behavioural phenotype in SAS.

**Supplementary Information:**

The online version contains supplementary material available at 10.1186/s11689-022-09426-0.

## Background

Functional haploinsufficiency of the *special AT-rich sequence-binding protein 2* (SATB2) gene located on chromosome 2q33.1 [1–3] is associated with craniofacial defects, most notably cleft palate [4, 5]. SATB2 gene variants are associated with a number of co-occurring manifestations (OMIM #612313), resulting in designation of a single clinically recognised syndrome [4] of SATB2-associated syndrome (SAS). SAS has an estimated frequency in undiagnosed developmental delay or intellectual disability of ~ .24 to .30% [6, 7]. Given the role of the SATB2 gene in neurodevelopment, the presentation of epileptiform discharges and diagnosis of seizures is particularly important, with an estimated prevalence of 93% and 42% respectively in SAS [8]. Regardless of variant (missense = 31%, nonsense = 24%, frameshift = 20%, intragenic deletion = 14% [9]) a consistent clinical phenotype is evident [6]. A diagnostic acronym has been adopted to enable the evaluation and surveillance of SAS [10]: severe speech anomalies (S); abnormalities of the palate (A); teeth anomalies (T); atypical behaviour, bone anomalies, and/or brain defects (B); and age of onset before 2 years (2).

Developmental delay and intellectual disability with delayed language acquisition are considered the hallmark universal characteristics of SAS [11]. In a recent clinical review of 121 school-age children and adults with SAS, 84% spoke fewer than ten words, and 42% evidenced completely absent speech [9]. Spoken language is not always a target for intervention in SAS and alternative means of expressive communication are possible with symbolic modalities such as sign language and picture communication systems [12], and therefore, communicative abilities of non-verbal individuals should not be underestimated. As such, a consideration of both receptive (the ability to comprehend and understand language) and expressive communication abilities (the ability to communicate thoughts, feelings, and needs with others) is important, as evidence in intellectual disability populations suggests stronger receptive communication abilities relative to expressive communication. Although there is some indication of relative strengths in receptive and non-verbal communication compared with spoken language in SAS [12], evidence is mixed when standardised assessments of communication are used. Direct assessment of communication profiles in 61 individuals with SAS elucidated both receptive and expressive language deficits [13], with only marginal gains in receptive vocabulary raw scores measured using the Test for Auditory Comprehension of Language–Fourth Edition [14] observed over time.

Although ‘behavioural issues’ (reported in 55% of individuals [15]) are one of the core diagnostic features of SAS, this broad categorisation is highly generalised and may obscure identification of specific behaviours. Based on clinical observation, autistic behaviours are described alongside a happy jovial disposition [7, 9, 11, 15]. Clinical case reports make reference to a ‘friendly’ disposition [16], hand stereotypies [17], repetitive interests, ‘inappropriate’ social behaviours (e.g. frequent touching and hugging, spontaneous bouts of laughter) and ‘autistic-like’ features [18]. Evidently, the description of behavioural characteristics in SAS varies markedly between individuals, and it is not clear how frequently these specific behavioural topographies occur in the larger SAS population.

Given the distinctive social profile evident in SAS, a differential diagnosis of Angelman syndrome (AS) is often considered in early infancy [4, 15]. AS is a clinically recognised syndrome characterised by frequent laughing and smiling, a happy demeanour, and absent speech [19, 20], and often accompanied by high rates of physical aggression [21] and clinically diagnosed autism [22]. Recently, whole exome sequencing analysis has identified the SATB2 gene as one of ten genes associated with an ‘AS-like’ phenotype in individuals with clinical features of AS of unknown genetic cause [23]. This highlights the clinical need to further delineate similarities and differences between these two syndromes in the present study.

Despite the significant phenotypic overlap with AS, SAS research to date has not utilised behavioural comparisons with analogous neurodevelopmental disorders to document the behavioural phenotype that characterises SAS. Similarly, autistic behaviours in SAS have not been comprehensively explored compared with a non-syndromal autism group. Autism is disproportionately diagnosed in males compared with females by a ratio of ~ 3:1 [24] that may reflect diagnostic overshadowing and sex- and gender-related differences in autism presentation in females [25]. It is important to note that such sex- and gender-related differences have not been reported in the current SAS literature [12]. Such group contrasts would be of clinical value to families and professionals, since qualitative differences in autism presentation exist between genetic syndrome groups associated with intellectual disability and autism [26, 27]. Evidently, there is a significant gap in knowledge about the SAS behavioural phenotype and a need to elucidate behavioural specificity in SAS using group-level cross-syndrome contrasts.

While clinical observations of aggressive behaviour (31%), hyperactivity (23%), agitation (> 45%), obsessive tendencies (~ 25%), sensory issues (~ 10%), and ‘difficult’ behaviour (11%) have been reported in SAS [7–10], specific behavioural topographies have rarely been explored. To date, only one study with children aged 2–16 years [28] has evaluated behaviour and emotion in SAS via use of a standardised measure—the Strengths and Difficulties Questionnaire (SDQ [29, 30]). Compared with normative data, children with SAS obtained higher SDQ scores for emotional problems, conduct problems, hyperactivity, and peer relationships and lower scores for prosocial behaviours. The SDQ impact score (as a measure of caregiver burden) was significantly higher in the SAS group compared with the normative group, with overall distress increasing with chronological age. However, the psychometric properties of the SDQ have not been established for populations with severe intellectual disability, and this study did not explore the behavioural profile or associated caregiver impact in adolescents or adults with SAS. Although there is some indication of change in behaviour over time based on clinical observation (e.g. tantrums, meltdowns, and aggressive outbursts in childhood with more physical acts of aggression towards others emerging in adolescence and adulthood [7]), these changes have never been explored using validated and standardised measures of specific behaviours.

To further delineate the behavioural phenotype of SAS, it is important to: (1) describe specific behaviours in association with age and take into account aspects relating to caregiver well-being, (2) utilise standardised measures with established psychometric properties and use in neurodevelopmental disorders associated with intellectual disability across all age groups, and (3) draw comparisons to contrast groups to characterise the specificity, nature, and severity of behaviours in SAS. In the largest standardised study of behaviours in SAS to date, this study aimed to:

1. Compare the profiles of specific topographies of behaviour and caregiver well-being scores between SAS developmental sub-groups: pre-school children, school-age children, and adolescents and adults with SAS, and explore associations between SAS participant characteristics and aspects relating to behaviour, autism, emotion, and caregiver well-being (*within-group analysis*).
2. Refine description of the behavioural phenotype in SAS through application of standardised measures appropriate for use in those with intellectual disability, comparing profiles in SAS to ability- and age-matched contrast groups at both subscale and item-level (AS and non-syndromal autism; *cross-syndrome analysis*).

## Methods

### Recruitment

Families caring for individuals with SAS were recruited in 2018–2019 via mailing list emails and social media research advertisements shared via closed groups affiliated with two support group organisations: the SATB2 Gene Trust UK and the international SATB2 Gene Foundation (USA). Families were included if they were the parent/caregiver of an individual with SAS aged 1 year and over diagnosed by a paediatrician, clinical geneticist, general practitioner, or neurologist and if the caregiver had proficient English language ability. Caregivers were invited to share genetic confirmation letters (where such a record of genetic information was available and families consented to genetic confirmation sharing).

The AS and non-syndromal autism groups were derived from a pre-existing dataset of participants held by the Cerebra Centre for Neurodevelopmental Disorders, University of Birmingham. These groups were originally recruited via the Angelman Syndrome Support Education and Research Trust and the National Autistic Society. Ethical approval was granted by Coventry Research Ethics Committee. Participants had received a diagnosis of AS or autism from a paediatrician, clinical geneticist, general practitioner or neurologist. As these data were collected as part of a larger questionnaire study for a historical dataset, genetic confirmation of diagnosis to determine molecular or chromosomal variants within the AS group cannot be reported. AS questionnaire responses were collected from 2003 to 2012, and non-syndromal autism questionnaire data were collected in 2007.

### Procedure

Parents/caregivers of children and adults with SAS completed an online survey created using LimeSurvey 2.00+ software [31]. The online survey included an information sheet, consent forms, and questionnaire measures (see *Measures*). Additional questionnaire measures were included in the SAS online survey that were not available for the AS and autism datasets. Therefore, cross-syndrome comparisons are not available for all measures included in the within-group SAS–only analysis (see *Measures* for further information).

### Participants

Data were excluded from three participants with SAS for whom a genetic diagnosis by a clinical professional was not reported. SAS genetic confirmation letters were available for 33 individuals. Overall, 81 participants with SAS were included in the SAS within-group analysis. To broadly explore age-related differences in SAS, the group was first divided according to three developmental sub-groups: pre-school children (aged 1–5 years), school-age children (aged 6–15 years), and adolescents and adults (aged 16 years and older).

Demographic and health-related information across developmental sub-groups is provided in Table 1. There were no significant differences between SAS developmental sub-groups for demographic characteristics such as gender or verbal ability, or health characteristics such as dental problems or cleft palate. Unsurprisingly, a difference in average self-help score, as measured by the Wessex Behavior Scale [32] as a proxy measure of level of ability, did approach statistical significance, with self-help score increasing with chronological age.

**Table 1:** Demographic and health related information across SAS developmental sub-groups and associated comparative analyses

	Developmental sub-group			Comparative analysis		
	Pre-school (PS; *n* = 30)	School-age (SA; *n* = 35)	Adolescents & adults (AA; *n* = 16)	Statistic^a^	*p* value	Post hoc test
***Demographics***						
*M* age; years (*SD*)	4.27 (*1.30*)	9.67 (*2.78*)	24.19 (*6.14*)	**186.018**	**< .001**	PS < SA < AA
Gender; *n* (% male)	15 (50.0)	16 (45.7)	12 (75.0)	3.964	.141	
Median self-help score^b, c^ (IQR)	5.00 (4.00–7.00)	6.00 (5.00–7.00)	6.50 (5.25–7.75)	*8.732*	*.013*	
Mobility^d^; *n* (% fully mobile)	25 (83.3)	35 (100.0)	15 (93.8)	-	.033	
Vision^c^; *n* (% normal)	23 (76.7)	31 (88.6)	13 (81.3)	-	.475	
Hearing^c^; *n* (% normal)	27 (90.0)	35 (100.0)	15 (93.8)	-	.141	
Speech^d^; *n* (% verbal)	6 (20.0)	13 (37.1)	7 (43.8)	3.420	.181	
***Health characteristics***						
*M* GDQ clinical signs (*SD*)	6.10 (*3.08*)	4.80 (*2.78*)	5.19 (*3.45*)	1.517	.226	
Eye problems^e^; *n* (% present)	5 (16.7)	7 (20.0)	0 (0.0)	-	.161	
Ear problems^e^; *n* (% present)	6 (20.0)	4 (11.4)	3 (18.8)	-	.670	
Dental problems^e^; *n* (% present)	11 (36.7)	20 (57.1)	7 (43.8)	2.800	.247	
Cleft palate^e^; *n* (% present)	4 (13.3)	1 (2.9)	1 (6.3)	-	.240	
GI problems^e^; *n* (% present)	8 (26.7)	2 (5.7)	2 (12.5)	-	.062	
Epilepsy^e^; *n* (% present)	6 (20.0)	7 (20.0)	0 (0.0)	-	.121	
Respiratory problems^e^; *n* (% present)	7 (23.3)	4 (11.4)	1 (6.3)	-	.326	
Skin problems^e^; *n* (% present)	7 (23.3)	11 (31.4)	4 (25.0)	-	.806	

Significant group differences highlighted in bold. Group difference italicised = test statistic approached statistical significance at *p* = .01 (deemed to approach statistical significance if *p* = .011 to .014). ^a^Test statistic for multiple-group comparison; Chi-square, ANOVA, or Kruskal–Wallis tests performed. Where test statistic is not reported, there were less than five expected values in cells, and Fisher’s exact test was performed. ^b^Noncategorical self-help scores were not normally distributed; therefore, Kruskal–Wallis test was conducted (median and IQR values reported). ^c^Data derived from Wessex Behavior Scale. ^d^Data derived from Background Information Questionnaire. ^e^Data derived from Health Questionnaire Part B; presence of health problem in the previous month (mild, moderate, and severe scores rated as present)

Participants within each syndrome group (SAS, AS, and autism) were ranked in ascending order. Each participant with SAS was matched to one participant with AS and one participant with non-syndromal autism, first according to self-help score (± 2 points) then chronological age (± 3 years). Following this matching strategy, some participants could not be matched within 2 points or 3 years, and 18 participants were excluded from the cross-syndrome analysis.

It is important to note, that there was a trend towards significance of these 18 excluded participants being older than those included in the cross-syndrome analysis (see Supplementary Materials 1; Additional File 1), but did not differ on any other demographic variables, such as gender or level of ability. Although gender is an important consideration in relation to autism profile, it was not possible to match according to gender in the present study. The existing autism dataset included only 42 females, and therefore, matching between the SAS and non-syndromal autism group would have been severely limited, leading to further participant exclusions (see *Limitations* for further comments). The included 63 participants were ability- and age-matched to 63 individuals with non-syndromal autism and 63 individuals with AS (see Table 2). There were fewer males with SAS or AS than non-syndromal autism and more mobile participants with SAS and autism than AS. There were also fewer verbal participants with SAS and AS than autism and more verbal participants with SAS than AS.

**Table 2:** Demographic characteristics of SAS, AS, and autism (aut) groups and associated comparative analyses.

	Neurodevelopmental group			Comparative analysis		
	SAS (*n* = 63)	AS (*n* = 63)	aut (*n* = 63)	Statistic^a^	*p* value	Post hoc test
Median age^*^; years (IQR)	7.07 (4.97–11.52)	8.73 (5.78–12.03)	7.77 (5.57–12.28)	1.821	.402	
Gender^b^; *n* (% male)	31 (49.2)	32 (45.7)	53 (84.1)	**19.832**	**< .001**	SAS, AS < aut
Median self-help score^* c ^(IQR)	6.00 (4.00–7.00)	6.00 (4.00–6.00)	6.00 (5.00–7.00)	4.031	.133	
Mobility^d^; *n* (% fully mobile)	59 (93.7)	44 (69.8)	58 (92.1)	**17.693**	**< .001**	AS < SAS, aut
Vision^c^; *n* (% normal)	52 (82.5)	56 (88.9)	61 (96.8)	6.822	.040	
Hearing^c^; *n* (% normal)	61 (96.8)	63 (100.0)	61 (96.8)	-	.548	
Speech^d^; *n* (% verbal)	20 (31.7)	4 (6.3)	47 (74.6)	**63.932**	**< .001**	AS < SAS < aut

Significant group differences highlighted in bold. *Noncategorical data were not normally distributed; therefore, Kruskal–Wallis test was conducted (median and IQR values reported). ^a^Test statistic for multiple-group comparison; Chi-square, ANOVA, or Kruskal–Wallis tests performed. Where test statistic is not reported, there were less than five expected values in cells, and Fisher’s exact test was performed. ^b^Gender information not available for two participants from AS group. ^c^Data derived from Wessex Behavior Scale. ^d^Data derived from Background Information Questionnaire

### Measures

Full descriptions of the measures used and their psychometric properties are presented in Table 3 [32–47]. Please note that for the majority of measures, higher scores indicate greater degree of difficulty, with the exception of the Mood, Interest, and Pleasure Questionnaire-Short Form (MIPQ-S) where higher scores are indicative of positive affect and increased interest and pleasure.

**Table 3:** Descriptive and psychometric properties of questionnaire measures (measures manual available via Oliver et al. [33])

Questionnaire measures	Description	Scoring information	Psychometric properties
Background Information Questionnaire	Reporting of gender, age, verbal ability, mobility, and diagnostic information of neurodevelopmental disorder (e.g. date of diagnosis, provision of diagnosis)	N/A	N/A
Wessex Behaviour Scale [32]	Proxy measure of level of ability in individuals with intellectual disability. Relevant *incapacities* and *speech* scales encompass subscales relating to incontinence, mobility, self-help, vision, hearing, speech, comprehension, and literacy.	Items are rated on a three-point scale from 1 (severe impairment) to 3 (no impairment). Self-help score is based on ability to independently: (1) wash, (2) feed, and (3) dress, with scores ranging from 3 (not able) to 9 (able).	Inter-rater reliability of the *incapacities* and *speech* scales originally reported by the authors range from 78% (self-help and literacy) to 92% (mobility) for both children and adults with intellectual disability.
Gastro-oesophageal Distress Questionnaire (GDQ [34]) ^a^	Assesses for behaviours indicative of gastro-oesophageal reflux. The questionnaire consists of 12 questions relating to behaviours in the last 2 weeks (e.g. 'cough, gag, or regurgitate?') and five questions relating to lifetime behaviours (e.g. 'Does the person you care for sleep sitting or propped up?').	The first 12 questions are rated on a five-point scale from 0 (not occurred) to 4 (more than once an hour) and the five lifetime questions are a combination of yes/no responses and four-point Likert scales. For each question, a score ≥ 2 or answer of yes is indicative of a cut-off for that item (equalling 1). Therefore, the total score is derived from the number of cut-off points obtained (maximum score of 17, ≥ 5 indicative of likely reflux).	N/A
Health Questionnaire (HQ [35]) ^a^	Measures the presence of 15 health conditions across the person’s lifetime (lifetime) and within the previous month (current). Of these 15 conditions, eight conditions that are highly prevalent in SAS are reported.	Associated severity scores can be calculated for both lifetime and current conditions on a four-point scale from 0 (never occurred) to 3 (severe). In this paper, only the presence of current health conditions in the SAS group (yes/no) are reported.	Good inter-rater reliability mean Kappa coefficient values are reported at item level for both lifetime (.72) and current health conditions (.76).
Social Communication Questionnaire (SCQ [36])	The lifetime version of the SCQ is used as a screening measure of autism characteristics and is validated in individuals aged 4 years and over. Formerly known as the Autism Screening Questionnaire, the 40 items are based on content from the Autism Diagnostic Interview.	Items are rated according to a yes/no response, with total scores ranging from 0 to 39 (question 1 relating to verbal ability is not included in total score calculation; a score of 0–33 is obtainable for non-verbal individuals). Three items are not grouped into subscales, the other 36 items are grouped according to *communication; reciprocal social interaction*; and *restrictive, repetitive, and stereotyped behaviour*. Cut-off scores of ≥ 15 and ≥ 22 are utilised as indicative of autism spectrum disorder and autism respectively.	Good diagnostic validity in school-age children with intellectual disability and pervasive developmental disorders, with sensitivity and specificity values of .92 and .62 respectively, when a cut-off score of ≥ 15 is utilised. Good concurrent validity reported with both the Autism Diagnostic Interview and the Autism Diagnostic Observation Schedule.
Repetitive Behaviour Questionnaire (RBQ [37])	Informant report measure of the occurrence of 19 observable operationally defined behaviours (e.g. hand stereotypy, organising objects, preference for routine) and their frequency during the previous month. Operationally defined definitions and further subscale information is provided by Moss et al. [38].	The frequency of behaviours is rated on a five-point scale ranging from 0 (never) to 4 (more than once a day). Items can be grouped into five subscales of repetitive behaviour: *stereotyped behaviour, compulsive behaviour, insistence on sameness, restricted preferences,* and *repetitive speech*. *Restricted preferences* and *repetitive speech* subscales are not calculated for individuals with limited verbal ability, therefore a maximum score of 76 can be obtained for verbal participants and 60 for non-verbal participants.	Spearman coefficients for inter-rater reliability range from .46 to .80 at item level with 73% of items above .60. Spearman coefficients test–retest reliability statistics range from .61 to .93 at item level with 52.6% of items above .80. Good concurrent validity is reported between the RBQ and the *restrictive, repetitive, and stereotyped behaviour* subscale of the SCQ (.60). Internal consistency is good at full-scale level (.80) and for the *stereotyped behaviour* and *compulsive behaviour* subscales (.70).
Challenging Behaviour Questionnaire (CBQ [39])	Informant report measure of the presence of self-injury, physical aggression, property destruction, and stereotyped behaviour in the last month on a yes/no basis.	A *self-injury severity* score can be calculated out of 14 based on the duration, response severity, and frequency of self-injury. Item scores are summed to provide an overall severity score, with higher scores denoting higher levels of self-injury severity. In this paper, stereotyped behaviour from the CBQ is not reported, as a more detailed description of stereotyped behaviour is provided by the RBQ *stereotyped behaviour* subscale.	Moderate to very strong Kappa coefficient values are reported for inter-rater reliability (.60 to .92), as well as good concurrent validity with the Aberrant Behavior Checklist (.56).
The Activity Questionnaire (TAQ [40])	Informant report measure comprising of 18 items relating to overactivity (e.g. ‘Does the person find it difficult holding still?’), impulsivity (e.g. ‘Does the person want things immediately?’), and impulsive speech (e.g. ‘Does the person often talk excessively?’).	Behaviour frequency is rated according to a five-point scale ranging from 0 (never/almost never) to 4 (always/almost all of the time). Items are grouped according to three subscales, with higher scores depicting greater behavioural severity: *overactivity* (0–36), *impulsivity* (0–24), and *impulsive speech* (0–24). *Impulsive speech* is not calculated for non-verbal participants.	Good mean item-level correlation coefficient values have been reported for both inter-rater reliability (.56) and test–retest reliability (.75). Inter-rater and test–retest reliability statistics are also good at both subscale and total score level (≥ .70).
Mood, Interest, and Pleasure Questionnaire—Short Form (MIPQ-S [41])	Measurement of affect, appropriate for use in individuals with intellectual disability. Six items correspond to mood (e.g. ‘In the last two weeks, do you think the facial expression of the person looked flat … ’), and six items correspond to interest and pleasure (e.g. ‘In the last two weeks, how interested did the person appear to be in his/her surroundings?’).	Behaviour frequency during the past 2 weeks are rated on a five-point scale from 0 (never) to 4 (all of the time), with subscale scores for *mood* and *interest and pleasure* ranging from 0 to 24. Higher subscale scores are indicative of more positive affect and higher levels of interest and pleasure.	This measure reports good internal consistency values for total score (.88), mood (.79), and interest and pleasure (.87), as well as good correlation coefficient values for both test–retest reliability (.97) and inter-rater reliability (.85).
Anxiety, Depression, and Mood Scale (ADAMS [42]) ^a^	Informant report measure comprising of 28 items that measure internalising states relating to anxiety, depression, and mood.	Items are rated on a four-point scale ranging from 0 (not a problem) to 3 (severe problem). Items are grouped according to five subscales: *manic/hyperactive behaviour, depressed mood, social avoidance, general anxiety,* and *compulsive behaviour*. In this paper, conservative subscale cut-off scores of ≥ 9 and ≥ 10 are utilised for *depressed mood,* and *generalised anxiety* respectively, as recommended by Hermans et al. [43].	This measure is specifically validated in older adults with intellectual disability (sensitivity = .80 to .82; specificity = .65 to .78), but also demonstrates good test-retest reliability in children and adults with intellectual disability aged 10-79 years (.81).
Hospital Anxiety and Depression Scale (HADS [44]) ^a^	This 14-item self-report measure of anxiety (e.g. ‘I get sudden feelings of panic’) and depression (e.g. ‘I look forward with enjoyment to things’) was originally developed for use in the general population but has since been used to measure caregiver well-being in a number of neurodevelopmental disorder studies (e.g. [45, 46]).	Items are rated on a four-point scale from 0 to 3, with higher scores denoting a greater severity of anxiety and depression symptomatology. A maximum score of 21 can be obtained on each *anxiety* and *depression* subscale, with cut-off scores ≥ 15 indicative of severe anxiety/depression and cut-off scores ≥ 8 indicative of mild symptomatology. In this paper, subscale cut-off scores of ≥ 8 are utilised, as recommended by Bjelland et al. [47].	Good specificity and sensitivity are reported for both anxiety (specificity = .78; sensitivity = .90) and depression (specificity = .79; sensitivity = .83) when a cut-off score of 8 is utilised. This measure also has good established concurrent validity (.60 to .80) when compared with standardised measures of anxiety and depression.

*N/A* not applicable; questionnaire is not a standardised behavioural assessment. ^a^Questionnaire measure is only available for within-group SAS developmental analysis; measures were not completed by AS and autism groups as part of previous cross-syndrome research database studies

### Data analysis

Data were analysed using Statistical Package for Social Sciences (SPSS), version 27. Within-group analyses broadly compared categorical data and cut-off scores between SAS developmental sub-groups using Chi-square analyses. Associations were also explored in the SAS group between participant characteristics and questionnaire subscales using Spearman rank correlation coefficients (two-tailed, *p* < .01) for continuous data and eta values for nominal by interval data (values closer to 1 indicating a higher degree of association). Across analyses, normality and homogeneity of variance were assessed via Shapiro–Wilk and Levene’s tests respectively; distributions were deemed to violate skewness and kurtosis when value/standard error statistics were greater than 1 *SD* (1.96).

In the cross-syndrome analysis, Chi-square tests were employed to compare categorical data between SAS, AS, and autism, and parametric one-way analyses of variance or nonparametric Kruskal–Wallis tests were conducted to compare continuous data between neurodevelopmental groups. Social Communication Questionnaire (SCQ) item-level analyses were calculated for 55 participants with SAS, 58 participants with AS, and 60 participants with autism (aged 4 years and over), and Repetitive Behaviour Questionnaire (RBQ) item-level analyses for four verbal items (*questions, echolalia, attachment people,* and *conversation*) were not calculated for the AS group. Non-verbal participants were not included in The Activity Questionnaire (TAQ) *impulsive speech*, RBQ *restricted preferences*, or RBQ *repetitive speech* developmental sub-group and cross-syndrome comparisons, as these subscales are not suitable for individuals with limited verbal ability.

Significant group differences were interrogated with the appropriate categorical (one-way Chi-square test), parametric (independent *t* test), or nonparametric (Mann–Whitney *U* test) post hoc analyses. Given the moderate sample sizes, exact rather than asymptotic significance tests were employed. To minimise the likelihood of type 1 errors, significant group difference alpha values were set at *p* < .01 to account for multiple-group comparisons. To prevent overreliance on statistical significance in the interpretation of the data, non-significant group differences were explored using Bayesian analyses to determine the degree of ‘commonality’ between groups (as outlined by Surtees et al. [48]). Such analyses are of clinical importance when considering similarity of the SAS phenotype to well-delineated neurodevelopmental groups. A Bayes Factor (*BF*_01_) is used to quantify support for the null hypothesis (groups do not differ) over the alternative hypothesis (groups significantly differ). Bayesian approaches do not rely on arbitrary cut-offs to establish ‘significance of commonality’, but in line with guidelines proposed by Jeffreys [49], a *BF*_01_ ≥ 3 provides ‘moderate and greater’ evidence in favour of the null hypothesis.

Post hoc group differences presented within the main text predominantly consider SAS–AS and SAS–autism comparisons in line with the study aims outlined. However, post hoc cross-syndrome analysis on AS–autism comparisons at both subscale and item-level is provided in the supplementary materials (see Supplementary Material 2 and 3; Additional File 1). Full statistical analysis of post hoc group differences at item level for the SCQ (SAS–AS, SAS–autism, AS–autism) are also provided within this supplementary information.

## Results

### Within-group SAS–only analysis

To determine the clinical utility of dividing the SAS group according to three developmental sub-groups, categorical data and cut-off scores were compared between pre-school children, school-age children, and adolescents and adults as presented in Table 4. There were no significant differences between developmental sub-groups for any measures of behavioural, autism, or emotional characteristics or on the Hospital Anxiety and Depression Scale (HADS) measure of caregiver well-being. The number of caregivers reaching clinical cut-off scores for anxiety was high across the pre-school children (76.7%), school-age children (62.9%), and adolescents and adults sub-groups (62.5%). The presence of self-injury and aggression was markedly high across all developmental sub-groups, as were the number of individuals meeting SCQ cut-off scores ≥ 15 (pre-school children: 35.0%; school-age children: 54.3%; adolescents and adults: 68.8%) as a measure of autism spectrum disorder characteristics. It is important to note however that differences in reported presence of property destruction did approach statistical significance, with a trend towards increasing prevalence with chronological age.

**Table 4:** Categorical data and cut-off scores across SAS developmental sub-groups.

Questionnaire measures	Developmental sub-group			Comparative analysis	
	Pre-school (PS; *n* = 30)	School-age (SA; *n* = 35)	Adolescents & adults (AA; *n* = 16)	Statistic^a^	*p* value
***Behavioural characteristics***					
CBQ self-injury; *n (%)*	12 (40.0)	14 (40.0)	8 (50.0)	.527	.802
CBQ *hit self with body*^b^; *n (%)*	9 (75.0)	7 (50.0)	4 (50.0)	-	.410
CBQ *hit self against object*^b^; *n (%)*	8 (66.7)	3 (21.4)	2 (25.0)	-	.058
CBQ *hit self with object*^b^; *n (%)*	2 (16.7)	0 (00.0)	2 (25.0)	-	.143
CBQ *bites self*^b^; *n (%)*	5 (41.7)	8 (57.1)	6 (75.0)	-	.377
CBQ *pulls self*^b^; *n (%)*	5 (41.7)	5 (35.7)	3 (37.5)	-	1.000
CBQ *rubs/scratches self*^b^; *n (%)*	3 (25.0)	6 (42.9)	3 (37.5)	-	.661
CBQ *inserts objects*^b^; *n (%)*	2 (16.7)	2 (14.3)	2 (25.0)	-	.857
CBQ aggression^c^; *n (%)*	21 (70.0)	26 (74.3)	14 (87.5)	3.113	.253
CBQ property destruction^c^; *n (%)*	9 (30.0)	19 (54.3)	11 (68.8)	*8.526*	*.013*
***Autism characteristics*** ^d^					
SCQ cut-off score ≥ 15; *n (%)*	7 (35.0)	19 (54.3)	11 (68.8)	4.188	.130
SCQ cut-off score ≥ 22; *n (%)*	4 (20.0)	10 (26.6)	3 (18.8)	-	.767
***Emotional characteristics***					
ADAMS depressed mood cut-off score ≥ 9; *n (%)*	3 (10.0)	5 (14.3)	3 (18.8)	-	.772
ADAMS general anxiety cut-off score ≥ 10; *n (%)*	4 (13.3)	8 (22.9)	2 (12.5)	-	.553
***Caregiver well-being***					
HADS anxiety cut-off score ≥ 8; *n (%)*	23 (76.7)	22 (62.9)	10 (62.5)	1.680	.435
HADS depression cut-off score ≥ 8; *n (%)*	11 (36.7)	11 (31.4)	3 (18.8)	-	.495

Given that the adolescents and adults SAS sub-group was not sufficiently sized to explore age-related differences at a group level, exploratory correlational analyses and eta values were reported to determine significant associations between SAS participant characteristics and questionnaire subscale scores (see Table 5). Chronological age and level of ability (continuous variables) and gender and seizure presentation (nominal variables) were selected as participant characteristics to explore in association with behaviour, autism, emotion, and caregiver well-being subscale scores, given their established or anticipated relevance to the SAS behavioural phenotype.

**Table 5:** Spearman’s rank correlation coefficient and eta values for continuous questionnaire data outcomes in SAS

Questionnaire measures	Correlational analysis		Eta values	
	Chronological age	Self-help score	Gender (*M*:*F* = 43:38)	Seizures (*Y*:*N* = 13:68)
***Behavioural characteristics***				
CBQ self-injury severity score^a^	.088	− .102	.180	.076
TAQ impulsivity	.103	− .189	.124	.032
TAQ overactivity	− .129	− .423*	.217	.123
TAQ impulsive speech^b^	− .009	.147	.148	.139
***Autism characteristics***				
SCQ reciprocal social interaction^c^	.168	− .304	.230	.243
SCQ communication^c^	.297	− .349	.252	.180
SCQ restrictive, repetitive, and stereotyped behaviours^c^	− .037	− .234	.187	.057
RBQ stereotyped behaviour	.001	− .288	.257	.074
RBQ compulsive behaviour	.159	.180	.061	.037
RBQ insistence on sameness	.305	.141	.052	.008
RBQ restricted preferences^b^	− .091	− .014	.087	.035
RBQ repetitive speech^b^	.154	.066	.254	.138
***Emotional characteristics***				
MIPQ-S mood	.040	.188	.155	.259
MIPQ-S interest and pleasure	− .268	.100	.153	.189
ADAMS manic/hyperactive behaviour	.144	− .254	.263	.058
ADAMS depressed mood	.227	− .045	.224	.026
ADAMS social avoidance	.094	− .113	.137	.193
ADAMS general anxiety	.340	− .134	.478*	.019
ADAMS compulsive behaviour	.305	− .031	.115	.179
***Caregiver Well-being***				
HADS anxiety	− .142	− .161	.244	.091
HADS depression	− .179	− .260	.230	.257

*Moderate association (.40 to .59), **strong association (.60 to .79), ***very strong association (.80–1.00). ^a^CBQ data only calculated for participants showing self-injury (*n* = 34). ^b^Subscales only calculated for verbal participants (*n* = 26). ^c^SCQ only valid for individuals aged 4 years and over; 10 participants under the age of 4 years excluded from SCQ analyses (*n* = 71)

Overall, few significant associations were observed between participant characteristics and behavioural questionnaire subscale scores. A moderate negative association was found between *overactivity* subscale scores and level of ability (*r*_*s* =_ − .423, *p* < .001) and an association was found between gender and Anxiety, Depression, and Mood Scale (ADAMS) *general anxiety* subscale scores (*η* = .478). Given that eta value direction of association or statistical significance cannot be inferred, interpretation was supplemented using a post hoc group analysis. ADAMS *general anxiety* subscale scores were significantly higher in males compared with those in females (*U*(1) = 361.00, *Z* = -4.331, *p* < .001, *BF*_01_ = .000).

### Cross-syndrome analysis

To contrast the profile of behaviours evident in SAS with well-delineated AS and autism phenotypes, categorical data, subscale average scores, and cut-off scores for the SAS group as a whole were compared with ability- and age-matched AS and non-syndromal autism groups.

### Behavioural characteristics

**Fig. 1:**
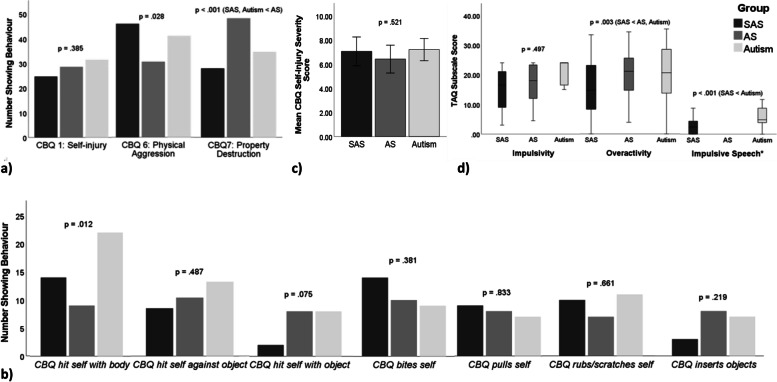
Bar graphs used to represent categorical data (**a**, **b**), histograms used to represent normally distributed continuous data based on the mean and *SD* (**c**), and boxplots used to represent non-normally distributed data based on the median and IQR (**d**). **a** Chi-square analyses comparing frequencies of self-injury, physical aggression, and property destruction (significant group differences at *p* < .01). **b** Chi-square analyses comparing frequencies of CBQ topographies of self-injury. **c** ANOVA analysis comparing CBQ self-injury severity scores between SAS, AS, and autism. Error bars represent 95% confidence intervals; ± (1.96 × standard error of the mean). **d** Kruskal–Wallis analyses comparing TAQ subscale scores between SAS, AS, and autism (significant group differences at *p* < .01). *AS group level comparisons were not conducted for the *impulsive speech* subscale due to small number of verbal participants (*n* = 3). Group level comparison conducted for verbal participants only (SAS: *n* = 20, autism: *n* = 46)

As shown in Fig. 1a–c, there were no significant differences between neurodevelopmental groups for: prevalence of self-injury, prevalence of specific topographies of self-injury, mean *self-injury severity* scores, or presence of physical aggression (prevalence of aggression was comparatively high across all groups). There were, however, significant differences relating to presence of property destruction, with higher rates observed in AS (84.7%). Differences were significant for both SAS–AS (46.8%; χ^2^(1) = 19.234, *p* < .001) and AS–autism group comparisons (59.0%; χ^2^(1) = 9.778, *p* = .002).

TAQ responses (see Fig. 1d) indicated no significant differences in *impulsivity* subscale scores between neurodevelopmental groups (SAS–aut: *U*(1) = 1882.00, *Z* = − .502, *p* = .618, *BF*_01_ = 7.176; SAS–AS: *U*(1) = 1794.00, *Z* = -.788, *p* = .433, *BF*_01_ = 5.195). However, scores on the *overactivity* subscale were comparatively lower in individuals with SAS than individuals with AS (*U*(1) = 1396.00, *Z* = -2.752, *p* = .006, *BF*_01_ = .138) and individuals with autism (*U*(1) = 1352.00, *Z* = -3.088, *p* = .002, *BF*_01_ = .076), despite decreased mobility in the AS group. The SAS group also evidenced a lower median *impulsive speech* subscale score than individuals with autism (*U*(1) = 207.50, *Z* = − 3.543, *p* < .001, *BF*_01_ = .011).

### Emotional characteristics

**Fig. 2:**
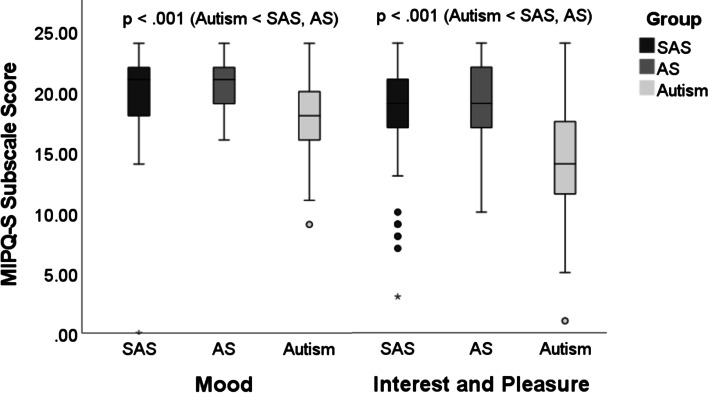
Boxplots used to represent non-normally distributed data based on the median and IQR. Mann–Whitney U analyses comparing MIPQ-S subscale scores between SAS, AS, and autism (significant group differences at *p* < .01). (★)  = significant outlier (not removed, nonparametric test conducted), • = outlier (not removed, nonparametric test conducted)

Both SAS and AS groups obtained higher *mood* (SAS: *U*(1) = 1293.00, *Z* = − 3.394, *p* = .001, *BF*_01_ = .289; AS: *U*(1) = 885.50, *Z* = − 5.307, *p* < .001, *BF*_01_ = .000) and *interest and pleasure* MIPQ-S subscale scores (SAS: *U*(1) = 985.00, *Z* = − 4.887, *p* < .001, *BF*_01_ = .000; AS: *U*(1) = 865.00, *Z* = − 5.835, *p* < .001, *BF*_01_ = .000) than individuals with autism (see Fig. 2). Median subscale scores were convergent for SAS and AS, and highly convergent in relation to *interest and pleasure* when *BF*_01_ values were scrutinised (*mood*: *U*(1) = 1570.00, *Z* = − 1.912, *p* = .056, *BF*_01_ = .661; *interest and pleasure*: *U*(1) = 1887.50, *Z* = − .325, *p* = .747, *BF*_01_ = 5.001). It is important to note, not all individuals with SAS evidenced comparatively high *interest and pleasure* scores, as eight individuals with SAS obtained scores ≤ 10 (see Fig. 2).

### Autism characteristics

**Fig. 3:**
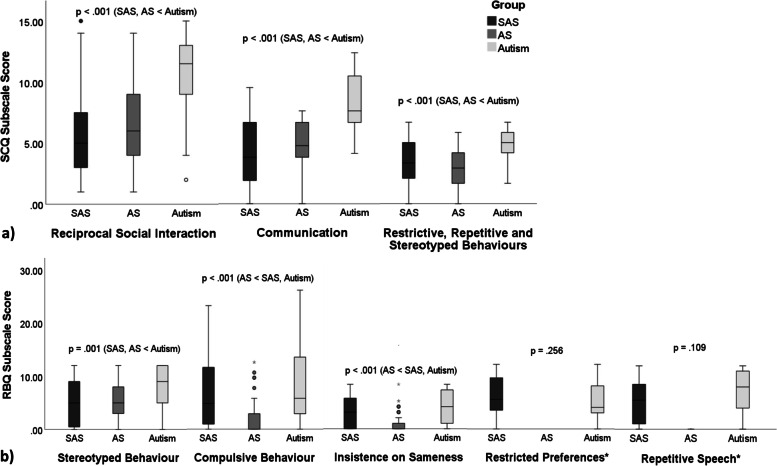
Boxplots used to represent non-normally distributed data based on the median and IQR **a**) Mann-Whitney U analyses comparing SCQ subscale scores between SAS, AS and autism (significant group differences at *p* < .01). **b**) Kruskal-Wallis analyses comparing RBQ subscale scores between SAS, AS and autism (significant group differences at *p* < .01). * = AS group level comparisons were not conducted for the *restricted preferences* and *repetitive speech* subscales due to small number of verbal participants (n = 4). Group level comparison conducted for verbal participants only (SAS: n = 20, autism: n = 46). ★ = significant outlier (not removed, nonparametric test conducted), • = outlier (not removed, nonparametric test conducted).

Across all SCQ subscales (see Fig. 3a), the SAS group evidenced lower subscale scores than the autism group (*reciprocal social interaction*: *U*(1) = 459.50, *Z* = − 6.613, *p* < .001, *BF*_01_ = .000; *communication*: *U*(1) = 384.00, *Z* = − 7.059, *p* < .001, *BF*_01_ = .000; *restrictive, repetitive, and stereotyped behaviours*: *U*(1) = 862.00, *Z* = − 4.460, *p* < .001, *BF*_01_ = .000). There were no significant differences between SAS and AS across SCQ subscale scores (*reciprocal social interaction*: *U*(1) = 1416.50, *Z* = − .883, *p* = .380, *BF*_01_ = 5.239; *communication*: *U*(1) = 1425.50, *Z* = − .833, *p* = .407, *BF*_01_ = .236; *restrictive, repetitive, and stereotyped behaviours*: *U*(1) = 1420.00, *Z* = 1.016, *p* = .312, *BF*_01_ = 3.821).

Fewer individuals with SAS (≥ 15: 52.7%; ≥ 22: 17.8%) met clinical cut-off scores on the SCQ than individuals with autism (≥ 15 (100.0%): χ^2^(1) = 36.131, *p* < .001; ≥ 22 (67.1%): χ^2^(1) = 40.505, *p* < .001). There were however no significant differences between SAS and AS in relation to the number of individuals meeting SCQ clinical cut-off scores (≥ 15 (54.4%): χ^2^(1) = .031, *p* = 1.000; ≥ 22 (15.1%): χ^2^(1) = .313, *p* = .648). Post hoc AS–autism comparisons are presented in Additional File 1.

On the verbal subscales of the RBQ (see Fig. 3b), there were no significant differences between SAS and autism in relation to *restricted preferences* (*U*(1) = 378.50, *Z* = − 1.144, *p* = .256, *BF*_01_ = 2.788) or *repetitive speech* (*U*(1) = 345.50, *Z* = − 1.606, *p* = .109, *BF*_01_ = 1.312). In relation to *stereotyped behaviour*, individuals with SAS obtained lower average scores than individuals with autism (*U*(1) = 1323.00, *Z* = − 3.255, *p* = .001, *BF*_01_ = .028).

Both SAS and autism groups evidenced higher *compulsive behaviour* (SAS: *U*(1) = 937.00, *Z* = − 5.211, *p* < .001, *BF*_01_ = .000; autism: *U*(1) = 731.00, *Z* = − 6.114, *p* < .001, *BF*_01_ = .000) and *insistence on sameness* subscale scores (SAS: *U*(1) = 999.00, *Z* = − 4.955, *p* < .001, *BF*_01_ = .000; autism: *U*(1) = 695.50, *Z* = − 6.208, *p* < .001, *BF*_01_ = .000) than the AS group. Median subscale scores were however more convergent for SAS and autism in relation to *compulsive behaviour* (*U*(1) = 1784.50, *Z* = − .835, *p* = .406, *BF*_01_ = 5.031) and *insistence on sameness* (*U*(1) = 1494.50, *Z* = − 2.026, *p* = .043, *BF*_01_ = 1.109).

Overall, cross-syndrome autism characteristics as measured by the SCQ and RBQ at subscale level revealed significant differences between SAS and autism, which were convergent for SAS and AS (SCQ: *reciprocal social interaction; communication*; *restrictive, repetitive and stereotyped behaviours*; RBQ*: stereotyped behaviour*) and significant differences between SAS and AS, which were convergent for SAS and autism (RBQ*: compulsive behaviour, insistence on sameness)*.

### Item-level cross-syndrome analysis

To elucidate whether fine-grained similarities and differences in autism profile existed between neurodevelopmental groups, SCQ and RBQ item-level analyses were conducted.

### SCQ item-level analysis

SCQ item-level analyses are presented in Table 6. Within the SCQ *reciprocal social interaction* domain, individuals with SAS were more likely to be reported as evidencing impairment on nine items compared with individuals with AS (inappropriate facial expressions, eye gaze, social smiling, showing and directing attention, seeking to share enjoyment, quality of social overtures, range of facial expressions, interest in other children, response to other children’s approaches). Across four items (inappropriate facial expressions, showing and directing attention, seeking to share enjoyment, quality of social overtures), individuals with SAS were more likely to be reported as evidencing impairment compared with individuals with autism. On three items (range of facial expressions, interest in other children, response to other children’s approaches), there were no significant differences between SAS and autism. Individuals with SAS were however less likely to evidence impairment on two items (imaginative play with peers, group play) compared with individuals with AS and autism.

**Table 6:** Item-level analyses comparing SAS, AS, and autism across individual SCQ items (excluding participants under 4 years)

Item number	Item	Number scoring on individual item			Chi-square test		
		SAS (*n* = 55)	AS (*n* = 58)	Autism (*n* = 60)	χ^2 ⁑^	*p* value	Post hoc test
	*Reciprocal social interaction*						
9	Inappropriate facial expressions^b^	44	19	26	**27.351**	**< .001**	SAS > AS**, aut**
10	Use of other’s body to communicate	50	45	48	3.949	.137	
19	Friends	27	31	44	8.065	.018	
26	Eye gaze^c, d^	43	25	37	**14.526**	**.001**	SAS > AS**
27	Social smiling^c, e^	44	15	40	**38.013**	**< .001**	SAS**, aut** > AS
28	Showing and directing attention^b, c^	44	25	25	**20.546**	**< .001**	SAS > AS**, aut**
29	Offering to share^f, g^	34	30	49	**13.224**	**.001**	aut > SAS*, AS**
30	Seeking to share enjoyment^b, c^	46	25	34	**19.229**	**< .001**	SAS > AS**, aut*
31	Offering comfort^f, c^	36	31	43	4.931	.088	
32	Quality of social overtures^b, c^	48	25	29	**26.042**	**< .001**	SAS > AS**, aut**
33	Range of facial expressions^f, c^	34	19	47	**27.307**	**< .001**	SAS*, aut** > AS
36	Interest in other children^b, g^	39	21	50	**30.522**	**< .001**	SAS**, aut** > AS
37	Response to other children’s approaches^b, g^	41	18	47	**32.785**	**< .001**	SAS**, aut** > AS
39	Imaginative play with peers^f, c^	7	45	54	**87.752**	**< .001**	AS**, aut** > SAS
40	Group play^b, c^	14	38	52	**48.159**	**< .001**	aut** > AS > SAS**
	*Communication*						
2^a^	Conversation	11	-	19	4.373	.061	
3^a^	Stereotyped utterances	11	-	39	-	.098	
4^a^	Inappropriate questions	6	-	26	1.813	.219	
5^a^	Pronoun reversal^b^	9	-	35	-	.103	
6^a^	Neologisms	9	-	34	**-**	.259	
20^a^	Social chat^f^	8	-	32	-	.080	
21	Imitation^b, c^	30	43	48	**10.794**	**.004**	aut > SAS*
22	Pointing to express interest^f, c^	38	33	46	6.147	.047	
23	Gestures^b, c^	43	34	42	4.651	.101	
24	Nodding to mean *yes*^b, c^	27	37	46	**10.355**	**.006**	aut > SAS*
25	Head shaking to mean *no*^b, g^	34	33	42	2.063	.359	
34	Imitative social play^b, c^	25	36	46	**12.860**	**.002**	aut > SAS**
35	Imaginative play^b, g^	24	44	47	**21.433**	**< .001**	AS**, aut** > SAS
	*Restrictive, repetitive, and stereotyped behaviour*						
7^a^	Verbal rituals	12	-	35	-	1.000	
8	Compulsions and rituals^h, c^	38	14	47	**39.711**	**< .001**	SAS > AS**, aut** > AS
11	Unusual preoccupations	26	24	43	**12.247**	**.002**	aut > AS*
12	Repetitive use of objects	33	48	42	5.659	.060	
13	Circumscribed interests	34	24	40	*8.557*	*.014*	
14	Unusual sensory interests	27	38	46	**9.559**	**.009**	aut > SAS*
15	Hand and finger mannerisms	34	43	52	**9.351**	**.009**	aut > SAS*
16	Complex body mannerisms^g^	21	26	45	**17.471**	**< .001**	aut > SAS**, AS*
	*Not in algorithm*						
17	Self-injury	25	28	36	2.781	.250	
18	Unusual attachment to objects	31	7	31	**28.419**	**< .001**	SAS**, aut** > AS
38	Attention to voice^b, c^	42	21	45	**25.447**	**< .001**	SAS**, aut** > AS

Significant group differences highlighted in bold. Group difference italicised = test statistic approached statistical significance at *p* = .01 (deemed to approach statistical significance if *p* = .011 to .014). *Post hoc analysis significant at *p* < .01, **post hoc analysis significant at *p* < .001. ⁑ Fisher’s exact test analysis conducted where value is not reported. ^a^Item only calculated for verbal participants (SAS; *n* = 14, autism; *n* = 41); Chi-square analyses only calculated for SAS–autism comparisons. ^b^Data missing for one participant in autism group. ^c^Data missing for one participant in AS group. ^d^Data missing for three participants in autism group. ^e^Data missing for four participants in autism group. ^f^data missing for two participants in autism group. ^g^Data missing for two participants in AS group. ^h^Data missing for one participant in SAS group

Within the *communication* domain, there were no significant differences between the three groups in relation to pointing to express interest, gestures and head shaking to mean *no*, and no significant differences between SAS and autism on six verbal items (conversation, stereotyped utterances, inappropriate questions, pronoun reversal, neologisms, social chat). However, significantly fewer individuals with SAS were reported to evidence impairment on four items on autism (imitation, nodding to mean *yes*, imitative social play, imaginative play) compared with individuals with autism.

Within the *restrictive, repetitive, and stereotyped behaviour* domain, more individuals with SAS and autism evidenced compulsions and rituals than individuals with AS. Across all three groups, there were no significant differences relating to repetitive use of objects. Across three items however (unusual sensory interests, head and finger mannerisms, complex body mannerisms), fewer individuals with SAS were noted to evidence difficulty than individuals with autism. There were no differences between groups relating to self-injury, but significantly more individuals with SAS compared with AS evidenced difficulty with unusual attachment to objects and attention to voice.

### RBQ item-level analysis

**Fig. 4:**
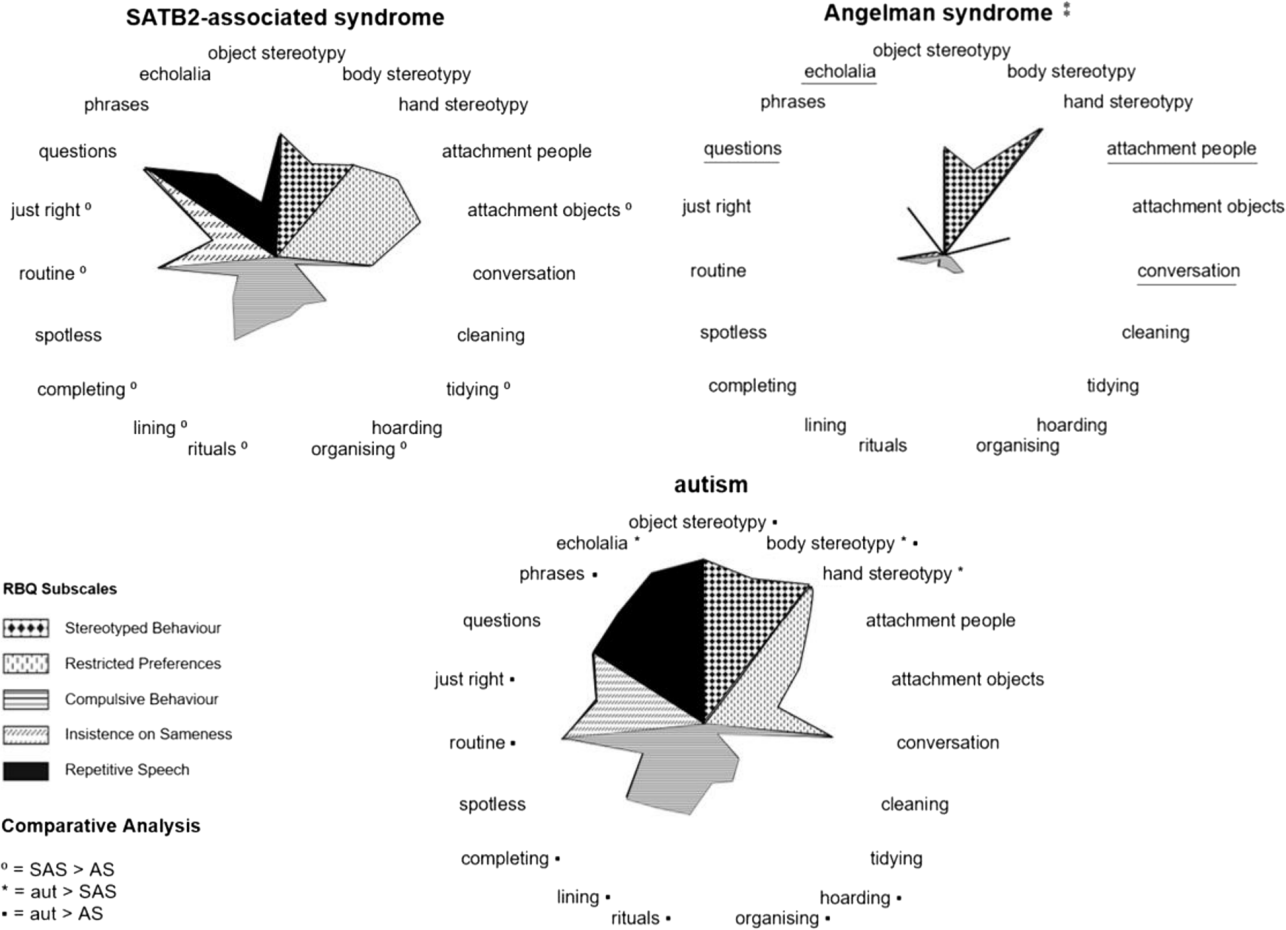
RBQ item-level analysis radar graphs comparing repetitive behaviour profiles between neurodevelopmental groups (significant group differences at *p* < .01, test statistics deemed to approach statistical significance at *p* = .011 to .014 are not reported). ⁑ Verbal items not calculated for the AS group (excluded items are underlined); verbal item analyses for SAS–autism comparisons (SAS; *n* = 20, autism; *n* = 47). AS missing data: rituals (*n* = 1), routine (*n* = 2), completing (*n* = 1), spotless (*n* = 1). Autism missing data: phrases (*n* = 1), rituals (*n* = 1), routine (*n* = 2), lining (*n* = 1), just right (*n* = 2), completing (*n* = 1), spotless (*n* = 1)

RBQ item-level radar graphs for each neurodevelopmental group are presented in Fig. 4. Mean item scores are presented to provide visual representation; however, nonparametric Kruskal–Wallis test statistics and post hoc Mann–Whitney *U* tests were conducted. Compared with the AS group, the SAS group obtained significantly higher median item scores on eight items: five items within the *compulsive behaviour* subscale (tidying: *U*(1) = 1501.50, *Z* = − 2.933, *p* = .003; organising: *U*(1) = 1425.50, *Z* = − 3.860, *p* < .001; rituals: *U*(1) = 1416.00, *Z* = − 3.687, *p* < .001; lining: *U*(1) = 1028.00, *Z* = − 5.707, *p* < .001; completing: *U*(1) = 1233.00, *Z* = − 4.811, *p* < .001), both items within the *insistence on sameness* subscale (routine: *U*(1) = 1158.00, *Z* = − 4.164, *p* < .001 and just right: *U*(1) = 1220.50, *Z* = − 4.879, *p* < .001), and one item within the *restricted preferences* subscale (attachment objects: *U*(1) = 1225.50, *Z* = − 3.960, *p* < .001).

Compared with autism, the SAS group obtained significantly lower median item scores on three items: two items within the *stereotyped behaviour* subscale (body stereotypy: *U*(1) = 1411.00, *Z* = − 2.936, *p* = .003; hand stereotypy: *U*(1) = 1371.00, *Z* = − 3.177, *p* = .001), and one item within the *repetitive speech* subscale (echolalia: *U*(1) = 217.00, *Z* = − 3.537, *p* < .001).

## Discussion

This study represents the largest sample of SAS behavioural data using standardised measures validated for individuals with intellectual disability, and is the first SAS study to adopt a cross-syndrome comparative approach to further delineate the profile of behaviours in this group. In summary, there were no significant developmental sub-group differences in cut-off scores or categorical questionnaire scores between pre-school children, school-age children, or adolescents and adults with SAS. Significant associations were found between higher rates of overactivity and lower self-help ability, and gender and general anxiety, with higher general anxiety scores reported for males with SAS compared with females. Cross-syndrome analysis revealed several distinct differences between SAS, AS, and non-syndromal autism groups, with SAS evidencing a behaviour profile characterised by comparatively low rates of property destruction, overactivity, impulsive speech, stereotyped behaviour, insistence on sameness, in contrast to positive affect and higher rates of interest and pleasure and compulsive behaviour. Although the SAS group obtained lower SCQ subscale scores than individuals with non-syndromal autism, fine-grained item-level analysis of both the SCQ and RBQ highlighted areas of significant difference, pinpointing a profile of repetitive behaviours, communication, and reciprocity with others that is distinct from both AS and autism.

### Behavioural characteristics: self-injury and aggression

In relation to the presentation of behaviours, 43% of children and adults with SAS evidenced self-injury, comparable to rates of self-injury reported in the wider non-syndromal autism literature (42% [50]). Cross-syndrome analysis in the present study did not elucidate any syndrome-related differences in relation to self-injury. SAS rates reported here are comparatively lower than rates of self-injury reported in Cri du Chat syndrome (77%), Cornelia de Lange syndrome (70%), and Smith-Magenis syndrome (93% [21]). Some may argue self-injury may not be a ‘hallmark’ behavioural characteristic of SAS when compared with Smith-Magenis syndrome for example [51]. However, a prevalence rate of 43% is still markedly high when compared with the general intellectual disability literature (12% [52]), and as such, the potential correlates of risk that may differentiate those with SAS who present with self-injury from those that do not warrant further investigation. Physical aggression was markedly high in SAS (77%) when compared with both the general intellectual disability literature (2–24% [53–55]) and rates of 20–31% that have previously been reported in the SAS literature [9, 10]. This may reflect methodological differences in data collection (informant report or clinical observation) and whether previous studies have utilised operationalised definitions of ‘aggressive’ behaviour (for example, taking into account differences between verbal, physical, and sexual aggression). In this study, a distinction was made between physical aggression (aggression directed towards others) and property destruction (aggression directed towards the environment), with lower rates of property destruction in SAS (47%) and autism (59%) compared with AS (85%).

### Behavioural characteristics: overactivity and impulsivity

At subscale level, individuals with SAS were reported by caregivers to evidence lower *overactivity* scores than individuals with AS or non-syndromal autism. Although overactivity has previously been reported in the SAS literature as characteristic of the behavioural phenotype [10], adopting a cross-syndrome comparison approach with AS, a well-delineated syndrome group with consistently high rates of overactivity [56, 57], has enabled us to weigh comparative overactivity ‘risk’ in SAS. When weighted against AS, overactivity may not be a defining characteristic of SAS. However, further examination of the data found relative to a maximum *impulsivity* subscale score of 24, moderately high median scores were reported across neurodevelopmental groups (SAS = 19, AS = 18.5, autism = 19), and there was notable variability in both *overactivity* and *impulsivity* TAQ subscale scores within the overall SAS group (*n* = 81; *overactivity* = 0–36, *impulsivity* = 3–24). Evidently, some individuals with SAS obtained high *overactivity* and *impulsivity* scores, but this is not meaningfully captured via group-level analyses.

Within-group analysis of the SAS cohort revealed a moderate association between higher rates of overactivity and lower self-help abilities. A similar relationship is reported in fragile X syndrome (FXS), a syndrome in which overactivity and impulsivity are particularly pronounced and described as a core behavioural phenotype [58]. In FXS, younger mental age is associated with increased likelihood of meeting diagnostic criteria for attention deficit hyperactivity disorder (ADHD) [59]. Future research in SAS should explore FXS-SAS cross-syndrome comparisons and item-level analyses of overactivity and impulsivity. Using FXS research as a theoretical model, SAS research should also consider potential associations with executive functioning deficits and the clinical utility of existing ADHD diagnostic criteria [60]. Furthermore, in syndrome groups such as FXS [61], as well as the non-syndromal autism literature [62], overactivity and impulsivity are identified as predictors of both the presence and persistence of self-injury and aggression. As such, the SAS literature would benefit from longitudinal research to delineate whether overactivity or impulsivity predict other severe forms of behaviours that challenge.

### Emotional characteristics

It is reassuring to note that no negative associations between *mood* or *interest and pleasure* subscale scores and chronological age were found, given that decline in levels of affect with age have been reported in other genetic syndrome groups [63, 64]. There are several challenges when exploring the mental health profiles of individuals with intellectual disability and autism, particularly the appropriateness of clinical measures and diagnostic criteria when individuals do not communicate using spoken language [65]. Although the present findings align with previous SAS literature regarding positive mood and affect [7, 11], and a highly convergent *interest and pleasure* profile with AS, a syndrome group where positive mood and affect are well-delineated [19, 20], eight individuals with SAS obtained markedly low *interest and pleasure* subscale scores. Given that there is an established relationship between low mood and the presentation of health problems in individuals with genetic syndromes associated with intellectual disability [66], professionals and caregivers should monitor whether any changes in health correlate with a noticeable change in mood. If changes are observed, it cannot be ruled out that pain may underlie some forms of behaviour [67]. Similarly to overactivity and impulsivity, low mood is a significant risk marker for self-injury [21, 62]. Future SAS behavioural phenotype research should therefore prioritise exploring the contribution of factors relating to an individual’s quality of life (e.g. untreated pain, health, affect, anxiety, and depression).

Such factors are difficult to measure in individuals who speak few or no words. Although the ADAMS was used in the present study as a measure of depression and anxiety (with good psychometric properties for validation in children and adults with intellectual disability; Table 3), several of the *depressed mood* items relate to sleep (e.g. easily fatigued) and several *general anxiety* items are also non-verbal indicators of pain (e.g. motor tension). The moderate association between gender and ADAMS *general anxiety* subscale scores, with higher scores evident in males compared with females, contradicts the consistent gender difference of higher prevalence of anxiety disorders in females compared with males reported in the general literature [68]. More research is needed to determine whether this general anxiety gender difference in SAS is replicable and observed across multiple contexts and whether the ADAMS is an appropriate measure in SAS. It is difficult to determine whether high ADAMS subscale scores accurately depicted depressed mood and anxiety, or were confounded by other biological characteristics of SAS that were not measured in the present study. Namely, sleep disorders that are particularly prevalent in young children [28], and painful dental and craniofacial abnormalities frequently reported in SAS [11]. Future behavioural research would benefit from the inclusion of sleep and pain measures that are validated in minimally verbal intellectual disability populations that do not violate multicollinearity assumptions with measures of depression and anxiety.

### Autism characteristics

Several autism characteristics were reported across all SAS developmental sub-groups at relatively high rates. Overall, 46% of children, adolescents, and adults with SAS met cut-off scores for autism spectrum disorder according to the SCQ. This is comparatively high when weighted against the prevalence of autism in other syndrome groups associated with autism and intellectual disability [27]. It is possible that the SCQ as a screening tool overestimates the diagnostic prevalence of autism characteristics in genetic syndrome groups [69], and as such further research is needed using ‘gold standard’ diagnostic measures (e.g. the Autism Diagnostic Observation Schedule [70]). However, there are clear clinical implications in adopting an SCQ item-level analysis approach as evidenced in other genetic syndrome groups [71], and a novel contribution to the literature in elucidating where the autism profile in SAS may deviate from the profile seen in non-syndromal autism. Compared with the non-syndromal autism group, individuals with SAS were *more likely* to evidence impairment on items relating to inappropriate facial expressions, showing and directing attention, seeking to share enjoyment, quality of social overtures, and less likely to evidence impairment on a number of play-focused and communicative items (imaginative play with peers, group play, imitation, imitative social play, imaginative play, and nodding to mean *yes*). From a clinical perspective, there are two key aspects to consider: (1) the effectiveness of targeted interventions based on the syndrome-related profile of autism characteristics in SAS; for example, the Joint Attention Symbolic Play Engagement and Regulation programme [72] could evidence particular promise in SAS, given that existing SAS play-based skills could be utilised to target relative areas of difficulty, namely the use of non-verbal social cues and joint attention skills. (2) Whether deficits in reciprocal social interaction are generalised or person specific; future research should aim to clarify whether non-verbal social difficulties in SAS are context specific, perhaps more closely aligning with a social anxiety profile or absence of social motivation as reported in other syndrome groups [73], or whether these difficulties are independent of social context, and do in fact align with a neurodevelopmental diagnosis of autism.

RBQ and SCQ item-level analyses pinpointed a clear discord between low-level stereotyped behaviour and high-level ritualistic and compulsive behaviours in SAS. Although stereotyped behaviours have been previously described in SAS case reports [17], this is the first cohort study to specifically delineate a ritualistic and compulsive repetitive behaviour profile in SAS. The wider non-syndromal autism literature highlights a distinction between lower-order and higher-order repetitive behaviours in relation to level of ability (e.g. stereotyped behaviours index low cognitive functioning and compulsive behaviours index high cognitive functioning [74]); however, the current SAS findings do not provide evidence of this distinction. The SAS group evidenced lower rates of hand and body stereotypies compared to non-syndromal autism, and higher rates of compulsive behaviours (e.g. tidying, organising, lining) compared with AS. These findings align with anecdotal reports that reference the need for objects to have their specific place and family members to adhere to fixed routines. Whether such compulsive behaviours are extrinsically mediated by social and environmental factors, or intrinsically driven by anxiety or cognitive factors such as executive functioning deficits, remains unclear. To inform behavioural intervention [75], functional analysis methodology would elucidate the extent to which intrinsic, extrinsic, and even anxiety-related factors underpin individual repetitive behaviour profiles in SAS.

### Limitations

Although this study has detailed several novel findings, utilising informant-report measures with enhanced specificity to explore behaviours in children and adults with SAS beyond subscale level, there are several methodological limitations to outline. It is important to emphasise the under-diagnosis of this recently recognised syndrome [7, 10]. This is the largest study of behavioural characteristics in SAS and makes a considerable clinical contribution to the literature; however, the skewed younger distribution of infants and young children presents an obvious threat to validity. Furthermore, exploratory genetic syndrome research with small samples is restricted by the effects of multiplicity and the family-wise error rate when performing multiple tests. Although a more conservative alpha value was employed, and a Bayesian analysis ‘weighted probability’ approach was adopted to supplement a strictly ‘frequentist’ approach [76], the false discovery rate of type 1 errors requires cautious consideration.

In favour of recruiting a representative sample to ensure external validity, an online international method of data collection was employed, relying exclusively on informant -report questionnaire measures. For brevity, the SCQ was used as a screening measure of autism and the Wessex Behavior Scale as a proxy measure of self-help abilities. Utilising a proxy measure undoubtedly oversimplified the developmental and intellectual profile of the SAS group, and the capacity of this study to adopt a more rigorous matching strategy based on level of ability. As such, the non-syndromal autism group may have lower adaptive functioning than reported in the wider autism population [77, 78]. Although the disproportionate male:female distribution evident in our non-syndromal autism group does reflect gender bias observed in the autism community [79], it is an obvious limitation that autism characteristics in SAS were explored without controlling for gender. Future social and behavioural models in SAS should consider the role of gender as a covariate or control variable.

Furthermore, several assessments were beyond the scope of the present study. As previously discussed, the potential contributions of untreated pain and poor sleep to the behavioural profile in SAS was not explored. Genotype–phenotype correlations were also not analysed within this study. Although participants had a confirmed genetic diagnosis of SAS, molecular genetic testing was not obtained for the majority of individuals. There is a largely consistent clinical phenotype in SAS independent of variant, but it is important to note genotype–phenotype analysis has elucidated some ‘phenotypic variation’ that may also apply to behavioural presentation, particularly in relation to larger contiguous deletions encompassing the SATB2 gene [80]. Language delay and seizure severity (both evident in SAS) are both identified as risk markers for behaviours that challenge in the wider neurodevelopmental literature [81, 82]. In SAS, more individuals with missense variants have absent speech and fewer individuals with nonsense variants have clinical seizures [9]. The present study was unable to account for the prevalence of larger intergenic variant subtypes as an extraneous variable associated with behaviours in SAS.

## Conclusions

Few significant associations were observed between participant characteristics and behavioural questionnaire subscale scores in the SAS group, except for a moderate negative correlation between overactivity and level of ability and higher *general anxiety* subscale scores in males compared with females. Overall, high levels of physical aggression (according to the Challenging Behaviour Questionnaire [CBQ]) and autistic behaviour (according to the SCQ) were reported by caregivers within the SAS cohort. Compared to age- and ability-matched AS and non-syndromal autism groups, the SAS cohort evidenced a comparative profile of positive affect and interest and pleasure as seen in AS, and a comparative profile of compulsive behaviour and insistence on sameness as seen in non-syndromal autism. At item-level, individuals with SAS evidenced a distinct repetitive behaviour profile characterised by low levels of stereotyped behaviours and high levels of compulsive behaviour (e.g. rituals) and insistence on sameness (e.g. attachment to objects). SCQ item-level analysis also revealed a distinct profile of autistic characteristics in SAS that differed from the non-syndromal autism group, with relative strength reported in relation to social behaviours, such as imitative social play and imaginative play, but relative difficulties in non-verbal social interactions, such as social overtures, directing attention and facial expressions. This SCQ item-level analysis approach uncovered variability in the autism profile and specific areas of strength and difficulty in SAS that were not apparent at subscale level. These findings have important clinical implications regarding the appropriateness and utility of existing autism and behavioural interventions in SAS, given the substantial qualitative differences in autism presentation in this syndrome group.

## Supplementary Information

**Additional file 1.** Group characteristics and associated comparative analyses for 18 participants in SAS group who could not be matched for inclusion in cross-syndrome analyses. Post hoc cross-syndrome statistics for AS-autism comparisons. Item-level post hoc comparisons across individual SCQ items (excluding participants under 4 years).

## Data Availability

The data that support the findings of this study are not publicly available. Due to the sensitive nature of personal data collected, participants were not asked to provide consent for data sharing as part of their research participation.

## Abbreviations

SAS: SATB2-associated syndrome
AS: Angelman syndrome
SATB2: Special AT-rich sequence-binding protein 2
SDQ: Strengths and Difficulties Questionnaire
GDQ: Gastro-oesophageal Distress Questionnaire
HQ: Health Questionnaire
SCQ: Social Communication Questionnaire
RBQ: Repetitive Behaviour Questionnaire
CBQ: Challenging Behaviour Questionnaire
TAQ: The Activity Questionnaire
MIPQ-S: Mood, Interest, and Pleasure Questionnaire Short Form
ADAMS: Anxiety, Depression, and Mood Scale
HADS: Hospital Anxiety and Depression Scale
SPSS: Statistical Package for Social Sciences
BF_01_: Bayes Factor
FXS: Fragile X syndrome
ADHD: Attention deficit hyperactivity disorder
