# Supplementary material for: The behavioural phenotype of SATB2-associated syndrome: a within-group and cross-syndrome analysis
Source: J Neurodev Disord. 2022 Mar 29;14:25. doi: 10.1186/s11689-022-09426-0 (PMC8966214; doi:10.1186/s11689-022-09426-0)
Supplement: Supplementary file 1 — Additional file 1. Group characteristics and associated comparative analyses for 18 participants in SAS group who could not be matched for inclusion in cross-syndrome analyses. Post hoc cross-syndrome statistics for AS-autism comparisons. Item-level post hoc comparisons across individual SCQ items (excluding participants under 4 years). [file 11689_2022_9426_MOESM1_ESM.docx]

| **Supplementary Materials 1. Group characteristics and associated comparative analyses for 18 participants in SAS group who could not be matched for inclusion in cross-syndrome analyses.** | | | | |
| --- | --- | --- | --- | --- |
|  | Group Statistics | | Comparative Analysis | |
|  | Included  (n = 63) | Excluded  (n = 18) | Statistic ^a^ | *p* value |
| Median age^*^; years  (IQR) | 7.07  (4.97-11.52) | 20.39  (5.59-27.72) | *344.00* | *.011* |
| Gender; n  (% male) | 32  (50.8) | 6  (33.3) | 1.714 | .284 |
| Median self-help score^*^ ^b^  (IQR) | 6.00  (4.00-7.00) | 6.50  (4.75-7.00) | 467.50 | .249 |
| Mobility ^c^; n  (% fully mobile) | 59  (93.7) | 16  (88.9) | **-** | .610 |
| Vision ^b^; n  (% normal) | 52  (82.5) | 15  (83.3) | - | 1.000 |
| Hearing ^b^; n  (% normal) | 61  (96.8) | 16  (88.9) | - | .212 |
| Speech ^c^; n  (% verbal) | 20  (31.7) | 6  (33.3) | .016 | 1.000 |

*Notes.* Significant group differences highlighted in **bold**. Group difference italicised = test statistic approached statistical significance at *p* = .01 (deemed to approach statistical significance if *p* = .011 - .014). * = non-categorical data were not normally distributed, therefore Mann-Whitney *U* values are reported with median and IQR statistics. a = test statistic depends on categorical or continuous data; Chi-square or Mann-Whitney *U* tests performed. Where test statistic is not reported, there were less than five expected values in cells and Fisher’s exact test was performed. b = data derived from Wessex Behavior Scale. c = data derived from Background Information Questionnaire.

| **Supplementary Materials 2. Post hoc cross-syndrome statistics for AS-autism comparisons.** | | | |
| --- | --- | --- | --- |
| Subscales/Items | AS-autism Post Hoc Analysis | | |
|  | Statistic | *p* value | BF_01_ |
| **Subscale Analysis** |  |  |  |
| ***Behavioural Characteristics*** |  |  |  |
| CBQ self-injury ^a^ | .131 | .856 | NA |
| CBQ self-injury severity score ^b^ | -1.098 | .276 | 3.078 |
| CBQ *hit self with body* ^b^ | **8.658** | **.005** | NA |
| CBQ *hit self against object* ^b^ | .213 | .795 | NA |
| CBQ *hit self with object* ^b^ | .053 | 1.000 | NA |
| CBQ *bites self* ^b^ | .287 | .782 | NA |
| CBQ *pulls self* ^b^ | .268 | .767 | NA |
| CBQ *rubs/scratches self*  ^b^ | .766 | .414 | NA |
| CBQ *inserts objects* ^b^ | .268 | .767 | NA |
| CBQ aggression ^c^ | 2.994 | .091 | NA |
| CBQ property destruction ^d^ | **9.778** | **.002** | NA |
|  |  |  |  |
| TAQ impulsivity ^e^ | 1729.00 | .269 | 4.416 |
| TAQ overactivity ^e^ | 1827.00 | .536 | 6.563 |
| TAQ impulsive speech | - | - | - |
|  |  |  |  |
| ***Autism Characteristics*** |  |  |  |
| SCQ reciprocal social interaction ^f^ | **565.50** | **< .001** | .000 |
| SCQ communication ^f^ | 1469.50 | .244 | .416 |
| SCQ restrictive, repetitive and stereotyped behaviours | **617.50** | **< .001** | .000 |
| SCQ cut-off score ≥ 15 ^f^ | **34.687** | **< .001** | NA |
| SCQ cut-off score ≥ 22 ^f^ | **47.189** | **< .001** | NA |
|  |  |  |  |
| RBQ stereotyped behaviour | **1318.50** | **.001** | -3.355 |
| RBQ compulsive behaviour ^f^ | **731.00** | **< .001** | -6.181 |
| RBQ insistence on sameness ^g^ | **695.50** | **< .001** | -7.215 |
| RBQ restricted preferences | - | - | - |
| RBQ repetitive speech | - | - | - |
|  |  |  |  |
| ***Emotional Characteristics*** |  |  |  |
| MIPQ-S mood ^e^ | **885.50** | **< .001** | 6.017 |
| MIPQ-S interest and pleasure ^e^ | **865.00** | **< .001** | 6.027 |
|  |  |  |  |
| **RBQ Item-Level Analysis ^h^** |  |  |  |
| RBQ *object stereotypy* | **1340.00** | **.001** | NA |
| RBQ *body stereotypy* | **1315.00** | **.001** | NA |
| RBQ *hand stereotypy* | 1749.00 | .216 | NA |
| RBQ *cleaning* | 1890.50 | .392 | NA |
| RBQ *tidying* | 1722.00 | .089 | NA |
| RBQ *hoarding* | **1536.00** | **.003** | NA |
| RBQ *organising* | **1456.50** | **< .001** | NA |
| RBQ *attachment people* | - | - | - |
| RBQ *questions* | - | - | - |
| RBQ *attachment objects* | 1570.00 | .025 | NA |
| RBQ *phrases* | **1133.00** | **< .001** | NA |
| RBQ *rituals* | **1221.50** | **< .001** | NA |
| RBQ *conversation* | - | - | - |
| RBQ *echolalia* | - | - | - |
| RBQ *routine* | **997.00** | **< .001** | NA |
| RBQ *lining* | **1108.00** | **< .001** | NA |
| RBQ *just right* | **799.00** | **< .001** | NA |
| RBQ *completing* | **933.00** | **< .001** | NA |
| RBQ *spotless* | **1522.50** | **.010** | NA |

*Notes.* Significant group differences highlighted in **bold**. NA = not applicable (BF_01_ not calculated for categorical or item-level data). Where test statistic is not reported = AS-autism group comparison not performed for verbal subscales/items due to small number of verbal participants in AS group (n = 4). a = CBQ self-injury data missing for three participants from AS group and one participant from autism group. b = CBQ self-injury data calculated only for participants showing self-injury (AS: n = 29, autism: n = 32). c = data missing for five participants from AS group and two participants from autism group. d = data missing for four participants from AS group and two participants from autism group. e = data missing for one participant from AS group. f = data missing for one participant from AS group and one participant from autism group. g = data missing for two participants from AS group and three participants from autism group. h = RBQ item-level missing data. AS missing data: rituals (n = 1), routine (n = 2), completing (n = 1), spotless (n = 1). Autism missing data: phrases (n = 1), rituals (n = 1), routine (n = 2), lining (n = 1), just right (n = 2), completing (n = 1), spotless (n = 1).

| **Supplementary Materials 3. Item-level post hoc comparisons across individual SCQ items (excluding participants under 4 years).** | | | | | | | | | | |
| --- | --- | --- | --- | --- | --- | --- | --- | --- | --- | --- |
| **Item Number** | **Item** | **Number Scoring on Individual Item** | | | **Chi-square Post Hoc Test** | | | | | |
|  |  | SAS  (n = 55) | AS  (n = 58) | autism  (n = 60) | SAS-AS | | SAS-autism | | AS-autism | |
|  |  |  |  |  | χ^2 ⁑^ | *p* value | χ^2 ⁑^ | *p* value | χ^2 ⁑^ | *p* value |
|  | *Reciprocal social interaction* |  |  |  |  |  |  |  |  |  |
| 9 | Inappropriate facial expressions ^b^ | 44 | 19 | 26 | **25.539** | **< .001** | **15.507** | **< .001** | 1.580 | .255 |
| 10 | Use of other’s body to communicate | 50 | 45 | 48 | NS | NS | NS | NS | NS | NS |
| 19 | Friends | 27 | 31 | 44 | NS | NS | NS | NS | NS | NS |
| 26 | Eye gaze ^c, d^ | 43 | 25 | 37 | **13.824** | **< .001** | 2.022 | .205 | 5.628 | .023 |
| 27 | Social smiling ^c, e^ | 44 | 15 | 40 | **32.361** | **< .001** | 1.441 | .278 | **21.957** | **< .001** |
| 28 | Showing and directing attention ^b, c^ | 44 | 25 | 25 | **15.457** | **< .001** | **16.868** | **< .001** | .026 | 1.000 |
| 29 | Offering to share ^f,^ ^g^ | 34 | 30 | 49 | .773 | .444 | **7.436** | **.010** | **12.796** | **< .001** |
| 30 | Seeking to share enjoyment ^b, c^ | 46 | 25 | 34 | **19.084** | **< .001** | **9.200** | **.004** | 2.199 | .193 |
| 31 | Offering comfort ^f, c^ | 36 | 31 | 43 | NS | NS | NS | NS | NS | NS |
| 32 | Quality of social overtures ^b, c^ | 48 | 25 | 29 | **23.244** | **< .001** | **18.868** | **< .001** | .326 | .582 |
| 33 | Range of facial expressions ^f, c^ | 34 | 19 | 47 | **9.111** | **.004** | 5.135 | .036 | **26.750** | **< .001** |
| 36 | Interest in other children ^b, g^ | 39 | 21 | 50 | **13.061** | **< .001** | 3.183 | .112 | **28.019** | **< .001** |
| 37 | Response to other children’s approaches ^b, g^ | 41 | 18 | 47 | **20.036** | **< .001** | .423 | .656 | **26.398** | **< .001** |
| 39 | Imaginative play with peers ^f, c^ | 7 | 45 | 54 | **49.349** | **< .001** | **73.416** | **< .001** | 4.810 | .033 |
| 40 | Group play ^b, c^ | 14 | 38 | 52 | **19.114** | **< .001** | **45.878** | **< .001** | **7.684** | **.007** |
|  | *Communication* |  |  |  |  |  |  |  |  |  |
| 2^a^ | Conversation | 11 | - | 19 | - | - | 4.373 | .061 | - | - |
| 3^a^ | Stereotyped utterances | 11 | - | 39 | - | - | - | .098 | - | - |
| 4^a^ | Inappropriate questions | 6 | - | 26 | - | - | 1.813 | .219 | - | - |
| 5^a^ | Pronoun reversal ^b^ | 9 | - | 35 | - | - | - | .103 | - | - |
| 6^a^ | Neologisms | 9 | - | 34 | - | - | **-** | .259 | - | - |
|  | | | | | | | | | | |
|  | | | | | | | | | | |
| **Item Number** | **Item** | **Number Scoring on Individual Item** | | | **Chi-square Post Hoc Test** | | | | | |
|  |  | SAS  (n = 55) | AS  (n = 58) | autism  (n = 60) | SAS-AS | | SAS-autism | | AS-autism | |
|  |  |  |  |  | χ^2 ⁑^ | *p* value | χ^2 ⁑^ | *p* value | χ^2 ⁑^ | *p* value |
| 20^a^ | Social chat ^f^ | 8 | - | 32 | - | - | - | .080 | - | - |
| 21 | Imitation ^b, c^ | 30 | 43 | 48 | 5.384 | .029 | **9.470** | **.003** | .600 | .502 |
| 22 | Pointing to express interest ^f, c^ | 38 | 33 | 46 | NS | NS | NS | NS | NS | NS |
| 23 | Gestures ^b, c^ | 43 | 34 | 42 | NS | NS | NS | NS | NS | NS |
| 24 | Nodding to mean *yes* ^b, c^ | 27 | 37 | 46 | 2.861 | .126 | **10.305** | **.002** | 2.427 | .151 |
| 25 | Head shaking to mean *no* ^b, g^ | 34 | 33 | 42 |  |  |  |  |  |  |
| 34 | Imitative social play ^b, c^ | 25 | 36 | 46 | 3.537 | .087 | **12.808** | **< .001** | 3.068 | .103 |
| 35 | Imaginative play ^b, g^ | 24 | 44 | 47 | **14.270** | **< .001** | **15.725** | **< .001** | .021 | 1.000 |
|  | *Restrictive, repetitive and stereotyped behaviour* |  |  |  |  |  |  |  |  |  |
| 7^a^ | Verbal rituals | 12 | - | 35 | - | - | - | 1.000 | - | - |
| 8 | Compulsions and rituals ^h, c^ | 38 | 14 | 47 | **23.369** | **< .001** | .950 | .392 | **33.869** | **< .001** |
| 11 | Unusual preoccupations | 26 | 24 | 43 | .397 | .573 | *7.115* | *.013* | **11.024** | **.001** |
| 12 | Repetitive use of objects | 33 | 48 | 42 | NS | NS | NS | NS | NS | NS |
| 13 | Circumscribed interests | 34 | 24 | 40 | NS | NS | NS | NS | NS | NS |
| 14 | Unusual sensory interests | 27 | 38 | 46 | 3.117 | .089 | **9.412** | **.003** | 1.787 | .224 |
| 15 | Hand and finger mannerisms | 34 | 43 | 52 | 1.974 | .225 | **9.395** | **.003** | 2.950 | .106 |
| 16 | Complex body mannerisms ^g^ | 21 | 26 | 45 | .773 | .444 | **15.907** | **< .001** | **9.958** | **.002** |
|  | *Not in algorithm* |  |  |  |  |  |  |  |  |  |
| 17 | Self-injury | 25 | 28 | 36 | NS | NS | NS | NS | NS | NS |
| 18 | Unusual attachment to objects | 31 | 7 | 31 | **24.816** | **< .001** | .255 | .709 | **21.180** | **< .001** |
| 38 | Attention to voice ^b, c^ | 42 | 21 | 45 | **17.766** | **< .001** | .000 | 1.000 | **18.378** | **< .001** |

*Notes*. Significant group differences highlighted in **bold**. Group difference italicised = test statistic approached statistical significance at *p* = .01 (deemed to approach statistical significance if *p* = .011 - .014). ⁑ = Fisher’s exact test analysis conducted where value is not reported. NS = post hoc analysis not performed; overall group analysis non-significant. a = item only calculated for verbal participants (SAS; n = 14, autism; n = 41); Chi-square analyses only calculated for SAS-autism comparisons. b = data missing for one participant in autism group. c = data missing for one participant in AS group. d = data missing for three participants in autism group. e = data missing for four participants in autism group. f = data missing for two participants in autism group. g = data missing for two participants in AS group. h = data missing for one participant in SAS group.
